# Educating the public and lawmakers about tobacco industry interference

**DOI:** 10.18332/tid/133366

**Published:** 2021-02-26

**Authors:** Barbara Schillo, Ann Boonn, Megan Arendt, Julie Bisbee

**Affiliations:** 1 Truth Initiative Schroeder Institute, Washington, United States; 2 Campaign for Tobacco-Free Kids, Washington, United States; 3 Action on Smoking and Health, Washington, United States; 4 Tobacco Settlement Endowment Trust, Oklahoma City, United States

**Keywords:** tobacco industry, monitoring, transparency, tobacco policy resources

Tobacco companies spend $9 billion annually in the US marketing and investing nearly $1 million each hour in promoting their products^1,2^. That investment is in addition to their ($28.3 million) large-scale, lobbying effort to weaken public health lawmaking and prolong the tobacco epidemic, which kills over 8 million people worldwide every year^3,4^. Combating this level of tobacco industry interference should be a priority for every tobacco control strategy. Fortunately, there are several resources and methods available to help combat industry meddling.

The first crucial resource to note is the result of a 1999 civil lawsuit by the U.S. Department of Justice against tobacco companies under the Racketeer Influenced and Corrupt Organizations Act (RICO)^5^. The RICO statute was intended to target organized crime. But, given the severe accusations against the tobacco industry, the U.S. Federal Court agreed to use RICO against them. In 2006, a Federal Judge delivered an opinion, finding that the defendants have engaged in and executed – and continue to engage in and execute – a massive fifty-year scheme to defraud the public, including consumers of cigarettes, in violation of RICO^6^.

Despite this ruling, research shows that the tobacco industry continues to find innovative ways to maintain and expand its interests. Tobacco executives have often referred to their main products as doubt: doubt of the harms, doubt of their influence on lawmakers, and doubt on the efficacy of public health laws. It is up to the public and civil society to combat that doubt, shine light on the bad acts of the tobacco industry, and allow policymakers to enact strong public health regulations^7^.

The Truth Initiative is a valuable resource for the tobacco control community. Truth Initiative tracks changes in US citizens’ perceptions of the tobacco industry and identifies ways to disrupt the tobacco industry’s marketing and reputation management efforts. Truth Initiative shares effective messages for youth and young adults to increase anti-tobacco industry perceptions and catalyze action against the industry^8^.

The report, *Spinning a New Tobacco Industry: How Big Tobacco is Trying to Sell a Do-Gooder Image and What Americans Think About It,* is an investigative piece that provides myriad examples to reveal tobacco industry tactics and information from Truth Initiative’s 2019 survey that assessed adult attitudes toward the industry^9^. This initial report illustrates the tobacco industry’s strong efforts to expand its portfolio and attract new customers while retaining existing ones. The industry is finding new ways to mitigate the limitations imposed by the Master Settlement Agreement and market to youth and young adults. Despite the tobacco industry’s extensive marketing strategies, Truth Initiative national polls report good news indicative of public distrust of the industry with 70% of adult US citizens viewing the tobacco and vaping industries unfavorably.

A second report published in 2020, *Seeing Through Big Tobacco’s Spin,* assesses youth and young adult perceptions of the tobacco industry and finds that they are even less trusting than adults^10^. In addition to believing that tobacco companies lie about how harmful their products are (92%) and believing them to be dishonest about the health effects of their products (69%), young people ‘did not buy’ that tobacco companies are part of the solution to end smoking (65%). Truth Initiative has a wealth of additional resources: from tobacco industry marketing to how tobacco companies exploit national crises to maintain their bottom lines.

The Campaign for Tobacco-Free Kids provides another valuable resource via their Industry Watch website that houses campaign materials specific to the tobacco industry and their bad acts in the US and globally^11^. Tobacco-Free Kids tracks tobacco companies’ political action committee contributions to federal members of Congress. It collects data on political contributions by company, by year, and by political party. They also provide resources around the RICO verdict and the legally mandated corrective statements that the tobacco companies must issue^12^. Tobacco-Free Kids has a wealth of fact sheets including those covering the tobacco industry’s general marketing, marketing to specific populations (i.e. children and Black Americans), general tobacco industry bad acts such as their misleading and disingenuous youth prevention programs, and interference with state and local policy^13-16^.

Tobacco-Free Kids monitors the tobacco industry to show how they continue to market to children and engage in strategies that harm public health. They analyze U.S. Federal Trade Commission reports on marketing expenditures by cigarette and smokeless tobacco companies to divulge extensive industry spending on tobacco product marketing^1^. Additionally, they track social media and other marketing to expose industry pricing activities like discounts that keep tobacco product pricing low; tobacco industry-sponsored events, such as the Reynolds American rooftop sessions with Rolling Stone; and the introduction of new and emerging tobacco products that attract youth and skirt FDA regulations.

Data show that one of the most effective methods to combat tobacco industry interference in lawmaking is to raise awareness. Many lawmakers are simply unaware that the tobacco industry influences many public health laws. It is up to the tobacco control community to educate them and the public through social media content, blogs, press releases, press events, letters to the editor and op-eds. The tobacco industry hopes the media misclassifies them as innocent bystanders or changed companies, but it is the job of the tobacco control community to ensure that they are accurately portrayed as the perpetrators of a global health epidemic. Effective earned media strategies can be deployed to take advantage of the valuable resources available from Truth Initiative, Tobacco-Free Kids, and Action on Smoking and Health’s 2020 Tobacco Industry Interference Index^17^. There are many ready-made reports, graphics and videos to utilize in state and local tobacco control work to strengthen the fight against big tobacco.

Additionally, there are concrete examples of effective internal policies and communications strategies that tobacco control advocates can reference. For example, exposing tobacco industry interference has been foundational to the Tobacco Settlement Endowment Trust (TSET)^18^ in Oklahoma. As an organization and state agency, TSET ensures that tobacco industry influence is constrained in their work, with their endowment investment policies excluding tobacco as part of their earnings. Furthermore, grantees and contractors must sign an affidavit that they do not receive tobacco funds.

TSET’s health communication interventions include paid and earned media under their Tobacco Stops With Me brand^19^. The brand’s campaigns include Not Ok, about the corrective statements from the tobacco industry, and a social campaign using the tobacco companies’ own words to show specific groups what tobacco companies really think of them. The corrective statements campaign amplified messages in a relevant and modern way using social media platforms, television and print advertisements, partner outreach, and toolkits available^20^.

With so many individuals and organizations in public health actively working to reclaim science, there is no better time than now to recognize tobacco industry interference. The tobacco industry continues to cast doubt on the facts and the evidence supporting sound, life-saving public health practices. The tobacco control community must highlight the pervasiveness of tobacco industry interference to neutralize its impact and save lives.
